# Airborne or Fomite Transmission for Norovirus? A Case Study Revisited

**DOI:** 10.3390/ijerph14121571

**Published:** 2017-12-14

**Authors:** Shenglan Xiao, Julian W. Tang, Yuguo Li

**Affiliations:** 1 Department of Mechanical Engineering, The University of Hong Kong, Hong Kong, China; liyg@hku.hk; 2 Clinical Microbiology, University Hospitals of Leicester NHS Trust, Leicester LE1 7RH, UK; jwtang49@hotmail.com; 3 Infection, Immunity, Inflammation, University of Leicester, Leicester LE1 7RH, UK

**Keywords:** norovirus, multi-agent simulation, fomite, airborne, outbreak analyses

## Abstract

Norovirus infection, a highly prevalent condition associated with a high rate of morbidity, comprises a significant health issue. Although norovirus transmission mainly occurs via the fecal-oral and vomit-oral routes, airborne transmission has been proposed in recent decades. This paper re-examines a previously described norovirus outbreak in a hotel restaurant wherein airborne transmission was originally inferred. Specifically, the original evidence that suggested airborne transmission was re-analyzed by exploring an alternative hypothesis: could this outbreak instead have occurred via fomite transmission? This re-analysis was based on whether fomite transmission could have yielded similar attack rate distribution patterns. Seven representative serving pathways used by waiters were considered, and the infection risk distributions of the alternative fomite transmission routes were predicted using a multi-agent model. These distributions were compared to the reported attack rate distribution in the original study using a least square methods approach. The results show that with some reasonable assumptions of human behavior patterns and parameter values, the attack rate distribution corresponded well with that of the infection risk via the fomite route. This finding offers an alternative interpretation of the transmission routes that underlay this particular norovirus outbreak and an important consideration in the development of infection control guidelines and the investigation of similar norovirus outbreaks in future.

## 1. Introduction

Norovirus was first reported in 1968 and has since placed a considerable disease burden upon economies and societies worldwide [1,2,3]. This pathogen, which is characterized by high excreted viral loads [4], remarkable environmental survivability [5], extreme infectivity [6] and short-term immunity [7], is now a leading cause of acute gastroenteritis across all age groups [1] and is responsible for approximately 685 million cases and 200,000 deaths annually worldwide [2,8].

Norovirus, a gastrointestinal virus, is thought to spread primarily by direct person-to-person contact or via foodborne, waterborne or environmental fomite routes [2]. Although no evidence suggests that this virus is transmitted via the airborne route [9], the importance of this route has been suggested by several published reports [10]. In terms of biological plausibility, the potential of airborne transmission of norovirus exists [11], based on the findings of virus-containing aerosol droplets produced by vomiting [12] and toilet flushing [13], the detection of dispersed norovirus in the air [14,15,16], and the successful infection of mice via intranasal instillation [17]. In terms of outbreak investigations, a few studies [18,19,20,21,22,23,24] have claimed to provide evidence for the airborne transmission.

To explore in greater detail the possibility of fomite versus airborne transmission of noroviruses, we reassessed the evidence in support of airborne transmission in a norovirus outbreak at a hotel restaurant in the UK in 1998 [21] by testing whether the alternative fomite transmission route could have led to a similar pattern of secondary cases. The original investigation concluded that the outbreak was due to airborne transmission because the authors assumed that only the airborne route could have resulted in an attack rate that exhibited an inverse relationship with the distance from the index patient [21]. Here, we demonstrate that with some waiters’ serving pathways and parameter values, a fomite transmission route could produce a similar pattern of secondary cases.

## 2. Methods

Information about the ventilation system settings and restaurant size were not available, so we could not perform computational fluid dynamics simulations [25,26] or multi-zone modelling [27,28] of fluid dynamics analyses to determine the infection risk due to the airborne route; we instead investigated whether the fomite route could produce similar spatial attack rate patterns. Specifically, we applied a multi-agent model to simulate the transmission of noroviruses from the index patient to susceptible hosts via surface contact and calculated the exposure doses and infection risks. Seven representative serving pathways of waiters were considered under different scenarios, using various values for two important parameters: the dose-response parameter on the mucous membranes of gastrointestinal tracts, $\eta_{m}$, and the viral load, $L_{0}$. For each scenario, least-squares fitting was used to compare the distributions of the predicted infection risks with those of the reported attack rates.

### 2.1. Outbreak

**Figure 1:**
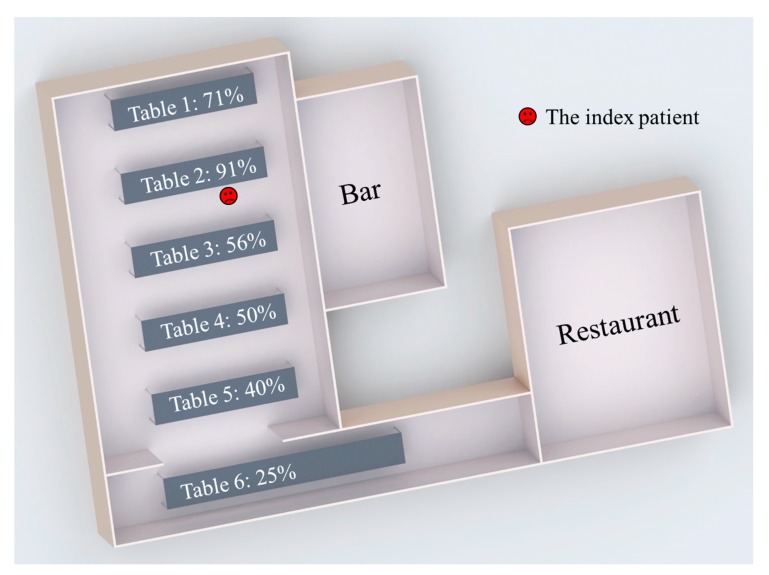
Floor plan of the site of the outbreak (restaurant) and the attack rates at different tables [21]. The location of the index patient is marked in red.

The norovirus outbreak occurred during an evening dinner at a hotel restaurant [21], as shown in Figure 1. The index patient was a woman who vomited onto the floor at 8:30 p.m. The vomiting was not projectile, and the vomitus was rapidly cleaned by a waiter. Since handwashing of the lady and the waiter might not be effective enough [29,30], we assumed that very small amounts of norovirus were initially carried by the index patient’s and the waiter’s hands at the beginning of the outbreak (Table S5). Because the meal was not disturbed by the vomiting episode, the following 90 min were assumed to be the potential exposure period.

In this study, the guests and waiters were identified as the study objects, and the investigation focused on the infection patterns of the guests. After the outbreak, 83 of 126 guests from six parties were interviewed, 52 of whom reported illness. As shown in Figure 1, the distribution of attack rates showed a statistically significant spatial pattern [21]. The attack rate was highest at Table 2, where the index patient was seated, while the rates at other tables decreased as the distance from Table 2 increased.

### 2.2. Multi-Agent Modelling Framework

The infection risks of various guests were predicted using a multi-agent modelling framework [27,28]. This framework considered 12 kinds of representative surfaces (Table S1), which were categorized into four types of materials: porous surfaces, non-porous surfaces, skin and mucous membranes. The four types of surfaces differed regarding properties such as transfer rates (Table S2) and first-order inactivation rates (Table S3).

**Figure 2:**
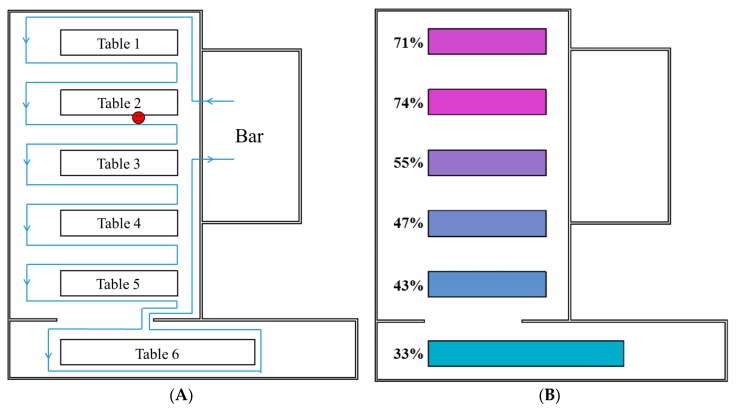

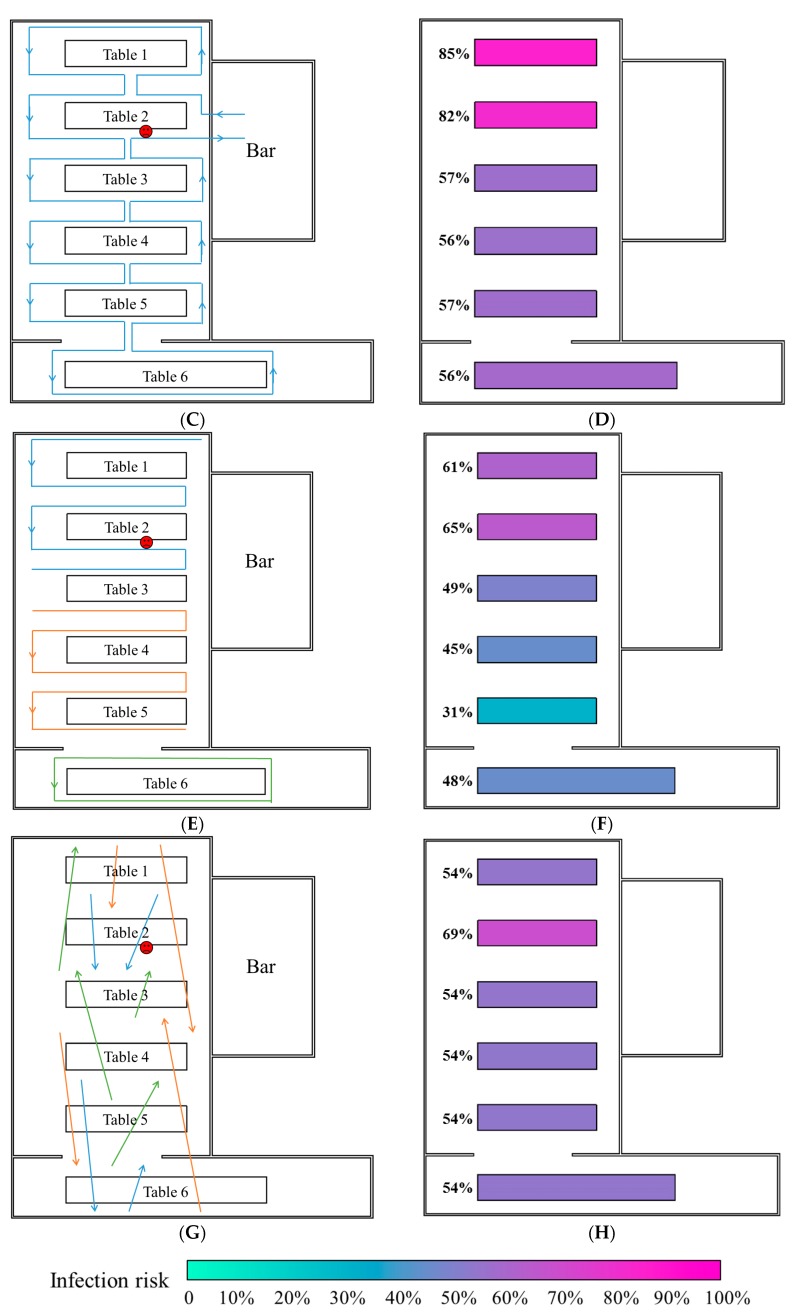
Waiters’ serving patterns and associated predicted infection risks of diners. (**A**) Serving Pathway 1. (**B**) Predicted average infection risk distribution (for 1000 simulations) via the fomite route at the end of the exposure period (Pathway 1). (**C**) Serving Pathway 3. (**D**) Predicted average infection risk distribution via the fomite route (Pathway 3). (**E**) Serving Pathway 5. (**F**) Predicted average infection risk distribution via the fomite route (Pathway 5). (**G**) Serving Pathway 7. (**H**) Predicted average infection risk distribution via the fomite route (Pathway 7). The dose-response parameter on mucous membranes, $\eta_{m}$ = 0.1415/genome copy, and the viral load, *L*_0_ = 3 × 10^8^ genome copies/g. The location of the index patient is marked in red. The different colors of tables represent different levels of infection risk.

Thirteen types of behavior, such as guests touching plates, guests shaking hands with others and waiters serving dishes, were assumed for both guests and waiters, and these assumed surface touching behaviors and their frequencies are summarized in Table S4. Because the six parties were discrete [21], it was assumed that guests did not shake hands with those at different tables. Seven types of pathways were considered regarding the behaviors of the waiters when serving dishes and wine. As shown in Figure 2A and Figure S1A, the waiters served guests at one table after another in anticlockwise and clockwise directions in Pathways 1 and 2, respectively. As shown in Figure 2C and Figure S1C, the waiters served guests at half of a table after another half in anticlockwise and clockwise directions in Pathways 3 and 4, respectively. As shown in Figure 2E and Figure S1E, the guests were divided into three groups of roughly equal size (Tables 1 and 2 and half of Table 3, the other half of Table 3 and Tables 4–6), and for every serving, each waiter served one group of guests in anticlockwise and clockwise directions in Pathways 5 and 6, respectively. As shown in Figure 2G, the waiters served guests randomly in Pathway 7.

After initializing the surfaces, people and behaviors, we applied a surface contamination model [27,28] to calculate the virus concentrations on different surfaces. The virus quantities on surfaces after one touching action only depend on the state before the action rather than the sequence of states that preceded it, which conforms to the definition of the Markov chain [31]. Therefore, every behavior consisting of a series of touching actions can be regarded as a discrete-time Markov chain, while environmental surfaces and hand surfaces can be regarded as different states in the Markov chain. The surfaces of the mucous membranes are special surfaces so the exposure doses to the mucous membranes were calculated via the surface contamination model. Furthermore, a dose-response relationship model [26,27,28,32] was used to predict the infection risk.

### 2.3. Least-Square Fitting

To evaluate the possibility of the seven types of waiters’ serving pathways, we compared the predicted infection risk of each pathway to the reported attack rates using least-square fitting methods. Specifically, we calculated the residual sum of squares (RSS) as a measure of the fit [33] for each pathway, and selected the probable pathway by maximizing fit (namely, minimizing RSS) [34].

As suggested by references [27,28], the dose-response parameter on the mucous membranes, $\eta_{m}$, and the viral load, $L_{0}$, strongly influence the infection risk distributions but might vary considerably among cases. According to the dose-response relationship model, we combined these two parameters as the product $\eta_{m}L_{0}$ to reduce the number of variables and defined the product as the dose effect of introducing 1 g of norovirus-containing particles with a viral load of $L_{0}$ to the mucous membranes. Based on the literature, we set a baseline scenario with a $\eta_{m}$ of 0.1415/genome copy [35] and a *L*_0_ of 3 × 10^8^ genome copies/g [4] and investigated different scenarios as the value of $\eta_{m}L_{0}$ varied from 10^5^ to 10^9^/g.

## 3. Results

### 3.1. Spatial Patterns of the Predicted Infection Risk Distributions

Figure 2B,D,F,H and Figure S1B,D,F present the average infection risk distributions of 1000 simulations involving the seven types of serving pathways at the end of the exposure period. In all simulations, the dose-response parameter on the mucous membranes, $\eta_{m}$, and the viral load, $L_{0}$, were set as the baseline values.

In this outbreak, the fomite transmission of norovirus between people occurred mainly via interactive behaviors such as hand shaking and waiters serving guests. Because it was assumed that the guests did not shake hands with those from other parties, hand shaking behavior merely influenced the regional distribution of the infection risk at each table. In contrast, the waiters’ serving behavior was not limited to single tables, which resulted in virus transmission between different tables. The waiter who initially carried the virus acted as a mobile virus source by which clean surfaces were contaminated, whereas other waiters acted as mobile media by which viruses were transmitted between surfaces. Thus, the overall infection risk distributions varied among the various serving pathway patterns (Figure 2 and Figure S1).

Table 2, where the index patient was seated, had very high average infection risks for all serving pathways because the other diners at that table had the opportunity to shake hands with the index patient, during which large amounts of noroviruses were transmitted. The infection risk distribution patterns at other tables were consistent with the waiters’ pathways. In Pathways 1 and 2 (Figure 2A and Figure S1A, respectively), wherein the waiters served guests at one table after another, the average infection risks of diners at each table (except Table 2) decreased according to the pathway direction (Figure 2B and Figure S1B, respectively). In Pathways 3 and 4 (Figure 2C and Figure S1C, respectively), the waiters served guests at half of a table after another half, resulting in a considerable diversity of infection risks among diners at the same table of Tables 3–5. Therefore, the average infection risks at Tables 3–5 were similar to that of Table 6. Because the diners at Table 1 were visited by waiters either very early (Pathway 3) or very late (Pathway 4), the average infection risk at that table was accordingly either very high (Figure 2D) or very low (Figure S1D). In Pathways 5 and 6 (Figure 2E and Figure S1E, respectively), wherein each waiter served the guests of one group, the infection risk decreased in the direction of the pathway in each group (Figure 2F and Figure S1F, respectively). In Pathway 7 (Figure 2G), the waiters served guests randomly, resulting in similar average infection risks at each table, except Table 2 (Figure 2H).

### 3.2. Fitness between Predicted Infection Risk and Reported Attack Rates

**Figure 3:**
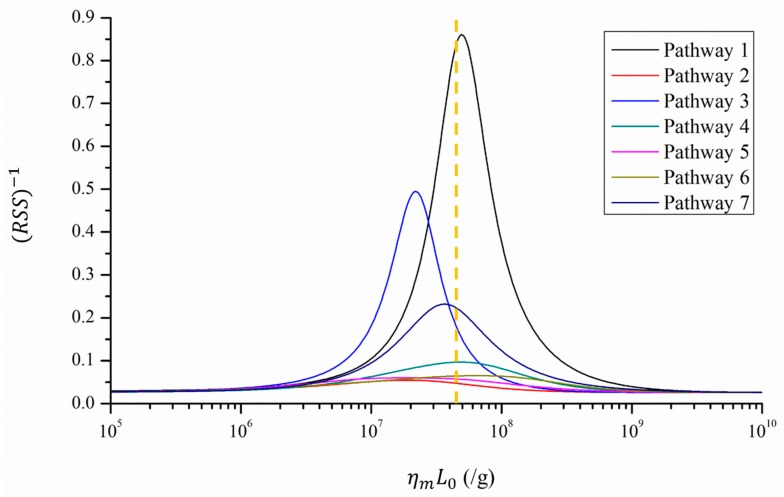
Fitness between the reported attack rates and predicted infection risks in different scenarios, using different values for the products of viral load and the dose-response parameter on mucous membranes, $\eta_{m}L_{0}$ (10^5^–10^9^/g). The fitness is inversely proportional to the value of the residual sum of squares (RSS) and is thus represented by (RSS)^−1^. Seven types of serving pathways are marked with seven colored lines. The orange dotted line indicates the baseline condition, where $\eta_{m}$ = 0.1415/genome copy and *L*_0_ = 3 × 10^8^ genome copies/g.

Figure 3 presents the fitness between the reported attack rates and predicted infection risks in different scenarios, while Table 1 lists the scenarios with the best fitness (the minimum RSS) for the seven types of serving pathways. According to the dose-response relationship model [26,27,28,32], the infection risk increased with the viral load, $L_{0}$, and the dose-response parameter of the mucous membranes, $\eta_{m}$. Excessively small or large values of these two parameters yielded correspondingly too low or high infection risks that deviated considerably from the attack rates. Therefore, the best fitness was reached with moderate values of $\eta_{m}L_{0}$, as shown in Figure 3 and Table 1.

Of the seven pathways, Pathway 1 exhibits the best fitness because the corresponding predicted infection risk distribution was qualitatively similar to that of the attack rates. As shown in Table 1, the infection risk was highest at Table 2 and decreased from Tables 1–6. As shown in Figure 3, the scenario with the best fitness is very similar to the baseline scenario, indicating that the index patient in this outbreak probably shed a viral load, $L_{0}$, similar to those reported by the literature [4], with a $\eta_{m}$ of 0.1415/genome copy [35].

## 4. Discussion

This re-analysis was conducted to investigate the hypothesis that airborne transmission was responsible for this previously described hotel norovirus outbreak [21]. The re-analysis was undertaken because hindsight and the subsequent investigation of many other outbreaks has called into question the proposed airborne transmission [21] in the absence of support from fluid dynamics analyses. The dispersion of airborne virus-containing aerosols is mainly determined by the airflow pattern in an indoor environment. The high level of complexity of these airflow patterns can be attributed to many factors, such as differences in mechanical ventilation settings. 

In some outbreaks, such as the largest nosocomial SARS outbreak in Hong Kong [26,27], fluid dynamics analyses demonstrated a decrease in the predicted average aerosol concentration as the distance from the index patient increased. However, in some other outbreaks, such as the first nosocomial MERS outbreak in South Korea [28], fluid dynamics analyses predicted a higher average aerosol concentration in distant wards (i.e., downstream wards) than in adjacent wards. Since the distributions of infections transmitted via airborne routes are mainly decided by the dispersion of airborne aerosols [25,26], it do not necessarily present an inverse relationship with the distance from the source. Thus, to determine the infection patterns attributable to the airborne route, airflow-dynamic studies that model virus-laden aerosols generated by an index patient should be performed where possible [25,26,27,28].

The results from this study indicate that given some reasonable assumptions about waiters’ serving pathways (e.g., Pathway 1), the infection risk attributable to the fomite route could exhibit an inverse relationship with the distance from the index patient, consistent with the attack rate distribution [21]. In other words, the attack rate distribution patterns in this outbreak [21] might have resulted from a fomite transmission route.

Aside from this outbreak investigation [21], the evidence presented by other researchers in support of the airborne transmission of norovirus [18,19,20,22,23,24] has been rather inconclusive. In a nosocomial outbreak in Toronto, reported by Sawyer et al. [18], housekeepers who visited or walked through the emergency room were more likely to become ill than those who did not encounter that area, and patients who visited the emergency room on 11 or 12 November were much more likely to develop infections than those who visited on 8 November. However, these characteristics could also be explained by fomite transmission because the surfaces surrounding the index patients would usually have been more contaminated [27,28] and surface contamination would be much more severe at a later stage of the outbreak than at an earlier stage [36]. 

Gellert et al. [19] suggested airborne transmission mainly to explain the lack of evidence of direct fecal contact among several cases of infection. However, as the fluorescence experiments [36] indicated, contaminants can spread to remote areas via the surface contamination network, wherein most contaminant transport between surfaces does not require direct contact with the original source. 

Several studies [20,21,22,23,24] have assumed that the index patients’ vomiting episodes provided sufficient evidence of airborne transmission. Nevertheless, as Booth suggested experimentally [12], vomiting may contaminate a wide range of surfaces that are often difficult to clean sufficiently, thus increasing the likelihood of further transmission of surface contamination. Moreover, Marks et al. [22] supported the possibility of airborne transmission because the time from exposure to illness decreased as the number of viral sources increased. However, as demonstrated by Lei et al. through simulations [36], an increase in the number of index patients could accelerate the transmission of surface contamination, thus increasing the accumulation of exposures and causing more rapid infection.

Regarding infectivity, even if norovirus infection is possible via inhalation of airborne droplets, the infection risk associated with the airborne route is not comparable to that of the fomite route. Viruses with high survivability on environmental surfaces usually have a higher exposure dose via the fomite route than that via the airborne route [27,28]. Moreover, unlike some respiratory viruses such as influenza [37], noroviruses should exhibit the same dose-response parameters for the airborne and fomite routes. The corresponding exposure site of norovirus, a gastrointestinal virus, is the gastrointestinal tract. The proposed airborne transmission would require inhaled aerosol droplets to be deposited in the upper respiratory tract and then be swallowed along with respiratory mucus [10,11]. 

According to the dose-response relationship [26,27,28,32,38] and assuming the same dose-response parameters, the fomite route with a higher exposure dose should be more important than the airborne route. Lei et al. [39] investigated the relative contributions of norovirus transmission routes in a flight norovirus outbreak, and predicted contributions of 96% and 4% for the fomite and airborne routes, respectively. Therefore, the contribution of the airborne route to norovirus transmission might be negligible when compared with the fomite route, and increased effort should be invested into controlling the transmission of fomite contamination.

This study had three limitations of note. First, detailed information about the ventilation systems and size of the restaurant are not available [21], so fluid dynamics analyses could not be performed. Therefore, the possibility of a long-range airborne route was not evaluated, and its relative importance versus the fomite route could not be accurately determined [27,28]. Second, due to the limited understanding of some of the parameters of norovirus transmission, some parameters in the model, such as the rate of transfer from hands to porous surfaces (Table S2), were estimated from those of other viruses or even bacteria. This might have reduced the accuracy of the results. Third, given the lack of real-time observation data, the model included many assumptions about the common surfaces and the actual behaviors and movements of the guests and waiters (Table S4), which might have affected the infection risk distributions shown in Figure 2 and Figure S1. In general, more detailed outbreak information, laboratory measurements and human behavioral observations would be required to provide a more accurate analysis of norovirus transmission in this outbreak.

## 5. Conclusions

This study re-examined the norovirus transmission routes in a previously reported hotel restaurant outbreak. With reasonable assumptions regarding the serving pathways of waiters and parameter values, the findings suggest that the observed spatial attack rate pattern could have resulted from the fomite transmission of norovirus (e.g., Pathway 1). This finding offers an alternative interpretation to the hypothesized airborne transmission route proposed by the original investigators of the outbreak. Furthermore, this study illustrates the need for a careful fluid dynamics analysis when evaluating the possibility of airborne transmission in an outbreak investigation. The results of this alternative transmission route analysis may assist the development of evidence-based infection control measures for norovirus outbreaks.

## Figures and Tables

**Table 1:** Comparison of the reported attack rates and predicted infection risks with the best fitness (minimum residual sum of squares, RSS) for the seven types of waiters’ serving pathways. The scenarios with the best fitness correspond to the highest points of the curves in Figure 3.

Parameter		Reported Data	Pathway						
			1	2	3	4	5	6	7
Minimum RSS		N.A.	1.16	18.26	2.02	10.33	16.67	15.35	4.32
$\eta_{m}L_{0}\;$^1^ (/g)		Unknown	10^7.69^	10^7.28^	10^7.34^	10^7.69^	10^7.26^	10^7.81^	10^7.56^
Average attack rate/infection risks at the six tables	Table 1	71%	73%	18%	74%	30%	48%	34%	52%
	Table 2	91%	76%	45%	73%	63%	55%	67%	67%
	Table 3	56%	58%	32%	43%	59%	35%	62%	52%
	Table 4	50%	49%	43%	42%	59%	33%	53%	51%
	Table 5	40%	46%	52%	42%	60%	19%	67%	51%
	Table 6	25%	35%	62%	44%	54%	33%	57%	51%
	Overall	63%	73%	18%	74%	30%	48%	34%	52%

^1^ $\eta_{m}L_{0}$ denotes the product of the dose-response parameter of mucous membranes and the viral load.

## Supplementary Materials

The following are available online at www.mdpi.com/1660-4601/14/12/1571/s1. Table S1: The material types and areas of surfaces. Table S2: Transfer rates between surfaces of different materials. Table S3: First-order inactivation rates at different sites. Table S4: Behavior frequencies and assumed touching surfaces during the behaviors. Table S5: Other parameters. Figure S1: Waiters’ serving patterns and predicted infection risks. (A) Waiters’ serving Pathway 2. (B) Predicted average infection risk distribution (for 1000 simulations) via the fomite route at the end of the exposure period (Pathway 2). (C) Waiters’ serving Pathway 4. (D) Predicted average infection risk distribution via the fomite route (Pathway 4). (E) Waiters’ serving Pathway 6. (F) Predicted average infection risk distribution via the fomite route (Pathway 6). The dose-response parameter on mucous membranes $\eta_{m}$ = 0.1415/genome copy and the viral load *L*_0_ = 3 × 10^8^ genome copies/g. The location of the index patient is marked in red. The different colors of tables represent different levels of infection risk.
