# Supplementary material for: Airborne or Fomite Transmission for Norovirus? A Case Study Revisited
Source: Int J Environ Res Public Health. 2017 Dec 14;14(12):1571. doi: 10.3390/ijerph14121571 (PMC5750989; doi:10.3390/ijerph14121571)
Supplement: Supplementary file 1 [file ijerph-14-01571-s001.pdf]

Supplementary

SI A. Parameter selection

Table S1. The material types and areas of surfaces.

Surface	Material Type	Area [a] (cm^2)	Data source
Napkin	Porous surfaces	1600	Calculated
Clothes		15,000	Calculated
Plate	Non-porous surfaces	78	Calculated
Spoon		20	Calculated
Fork		20	Calculated
Knife		20	Calculated
Glass		90	Calculated
Wine bottle		440	Calculated
Hand	Skin	100	Estimated from [1]
Finger contact area		3	Estimated from [2]
Non-mucosal regions of head and neck		1600	Calculated
Mucous membranes	Mucous membranes	10	Based on [3]

[a] Surface areas are effect areas that are commonly touched rather than the actual areas.

Table S2. Transfer rates between surfaces of different materials.

Donor Surface	Acceptor Surface	Transfer Rate	Data Source
Hand	Porous surface	80%	Estimated from [4]
Hand	Skin	50%	Assumed
Hand	Non-porous surface	12%	Estimated from [5]
Hand	Mucous membranes	36%	Estimated from [6]
Porous surface	Hand	3%	Estimated from [7]
Skin	Hand	50%	Assumed
Non-porous surface	Hand	11%	Estimated from [5]
Mucous membranes	Hand	0%	Assumed

Table S3. First-order inactivation rates at different sites.

Site	Value	Data Source
On porous surface	0.5/hr	Estimated from [8]
On skin	2.4/hr	Estimated from [9]
On non-porous surface	0.1/hr	Estimated from [10]

Table S4. Behaviour frequencies and assumed touching surfaces during the behaviours.

Behaviour	Executors	Frequency	Touching Surfaces	Data Source
Touching one's own non-mucosal regions of head and neck	All agents	13/h	Non-mucosal regions of head and neck	Estimated from [11]
Touching one's own mucous membranes	All agents	9/h	Mucous membranes	Estimated from [12]

Touching plates	Guests	30/h	Plates	Assumed
Touching spoons	Guests	30/h	Spoons	Assumed
Touching forks	Guests	30/h	Forks	Assumed
Touching napkins	Guests	10/h	Napkins	Assumed
Touching glasses	Guests	30/h	Glasses	Assumed
Touching clothes	Guests	6/h	Clothes	Assumed
Handshaking with others	Guests	0~3 times during the dinner	Hands	Assumed
Serving the dishes	Waiters	5 times during the dinner	Plates	Assumed
Serving the wine	Waiters	5 times during the dinner	Glasses	Assumed
Taking back the plates	Waiters	5 times during the dinner	Plates	Assumed
Taking back the glasses	Waiters	5 times during the dinner	Glasses	Assumed

Table S5. Other parameters.

Parameter	Description	Value	Data Source
T	Computational duration	90 minutes	Assumed from information from [13]
L_0	Virus concentration in vomitus	3×10^8 genome copies/g	Estimated from [14]
Q_{ih}	Virus quantities on the index patient's hand after vomiting	3×10^5 genome copies	Estimated from [9]
Q_{wh}	Virus quantities on a waiter's hand after he cleaned the vomitus	1.5×10^4 genome copies	Assumed
η_m	Dose response parameter on the gastrointestinal tract	0.1415/ genome copy	Estimated from [15]
ε_w	Hand washing efficiency	0.4	Estimated from [16]
N_{wd}	Number of waiters serving dishes	3	Assumed from the information from [13]
N_{ww}	Number of waiters serving wine	3	Assumed from the information from [13]
N_{ii1}	Number of interviewed ill guests in Table 1	5	Derived from information from [13]
N_{i1}	Number of interviewed guests in Table 1	7	Derived from information from [13]
N_{t1}	Number of the total guests in Table 1	8	Derived from information from [13]

N_{ii2}	Number of interviewed ill guests in Table 2	20	Derived from information from [13]
N_{i2}	Number of interviewed guests in Table 2	22	Derived from information from [13]
N_{t2}	Number of the total guests in Table 2	22	Derived from information from [13]
N_{ii3}	Number of interviewed ill guests in Table 3	14	Derived from information from [13]
N_{i3}	Number of interviewed guests in Table 3	25	Derived from information from [13]
N_{t3}	Number of the total guests in Table 3	25	Derived from information from [13]
N_{ii4}	Number of interviewed ill guests in Table 4	10	Derived from information from [13]
N_{i4}	Number of interviewed guests in Table 4	20	Derived from information from [13]
N_{t4}	Number of the total guests in Table 4	20	Derived from information from [13]
N_{ii5}	Number of interviewed ill guests in Table 5	2	Derived from information from [13]
N_{i5}	Number of interviewed guests in Table 5	5	Derived from information from [13]
N_{t5}	Number of the total guests in Table 5	6	Derived from information from [13]
N_{ii6}	Number of interviewed ill guests in Table 6	1	Derived from information from [13]
N_{i6}	Number of interviewed guests in Table 6	4	Derived from information from [13]
N_{t6}	Number of the total guests in Table 6	45	Derived from information from [13]

SI B. Supplemental figures

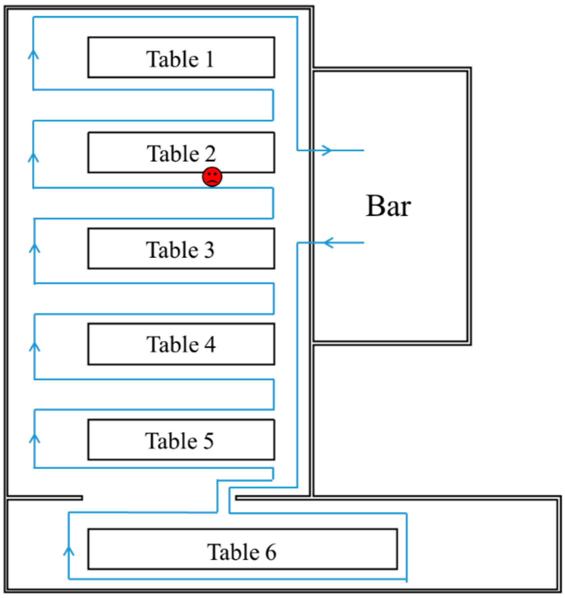
Table 1
Table 2
Table 3
Table 4
Table 5
Table 6
Bar

(A)

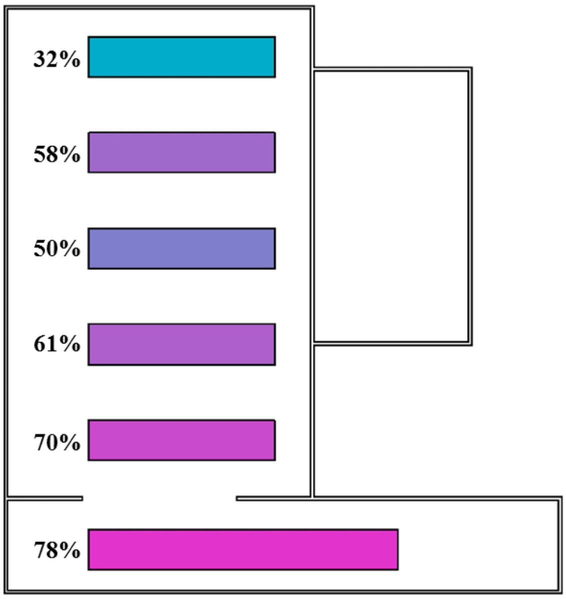
32%
58%
50%
61%
70%
78%

(B)

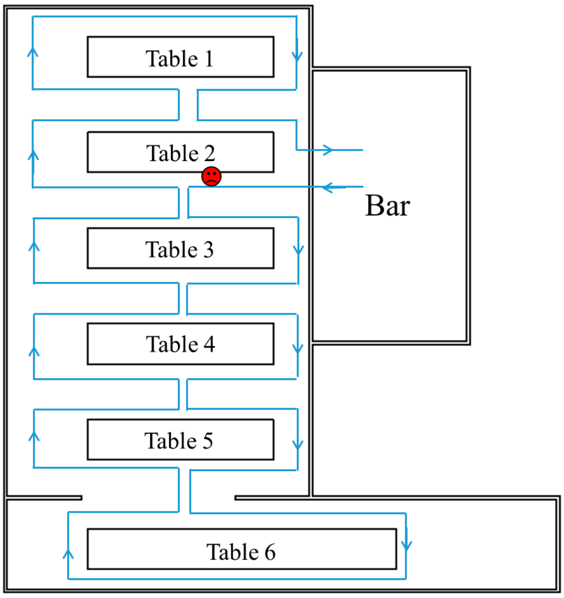
Table 1
Table 2
Table 3
Table 4
Table 5
Table 6
Bar

(C)

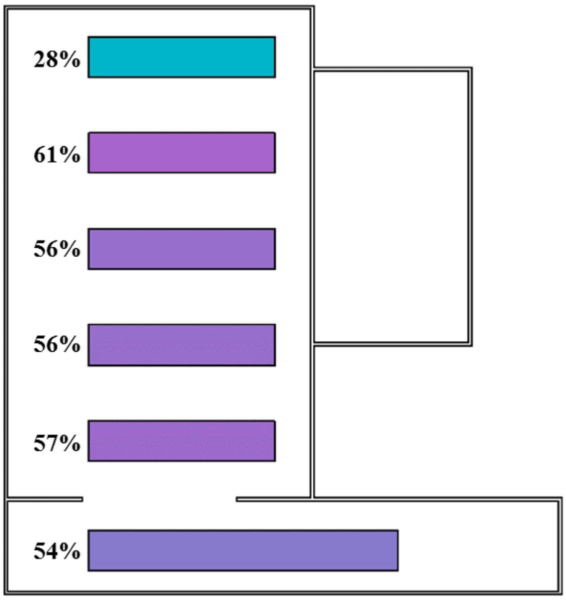
28%
61%
56%
56%
57%
54%

(D)

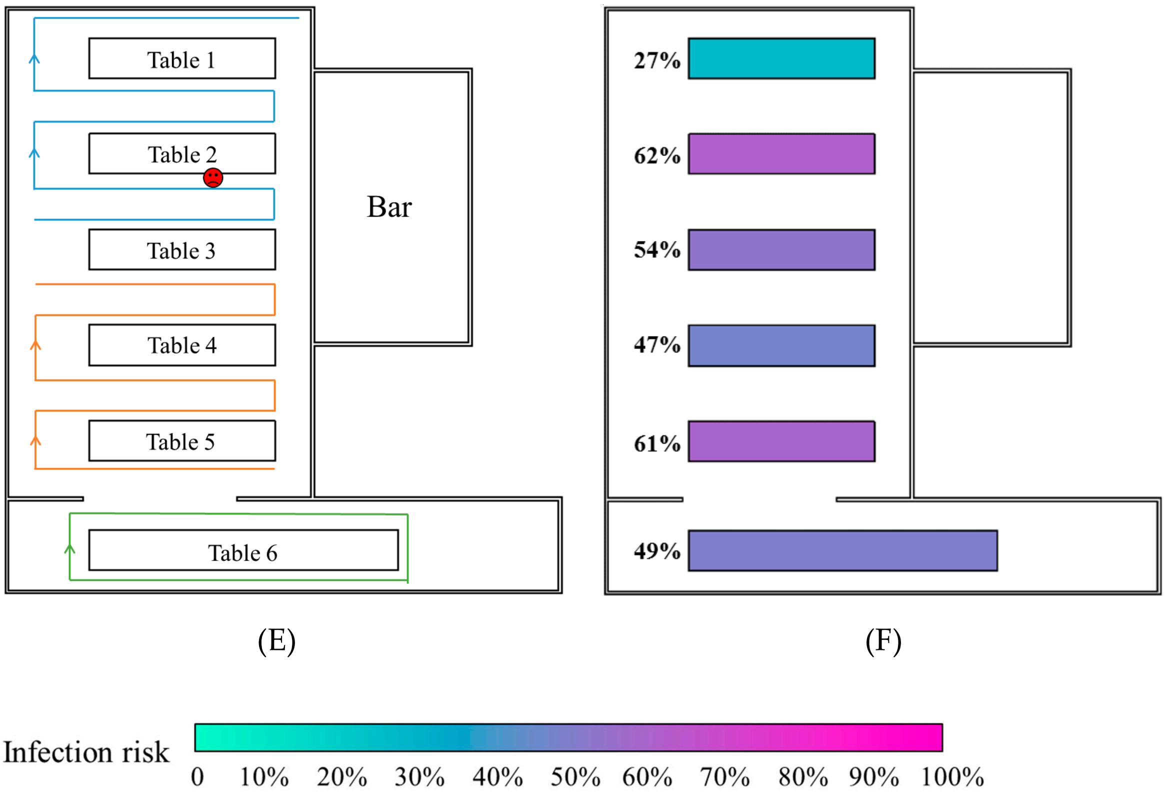

Figure S1. Waiters' serving patterns and predicted infection risks. (**A**) Waiters' serving Pathway 2. (**B**) Predicted average infection risk distribution (for 1,000 simulations) via the fomite route at the end of the exposure period (Pathway 2). (**C**) Waiters' serving Pathway 4. (**D**) Predicted average infection risk distribution via the fomite route (Pathway 4). (**E**) Waiters' serving Pathway 6. (**F**) Predicted average infection risk distribution via the fomite route (Pathway 6). The dose-response parameter on mucous membranes η_m = 0.1415/genome copy and the viral load L_0 = 3×10^8 genome copies/g. The location of the index patient is marked in red. The different colors of tables represent different levels of infection risk.

References

1. Lee, J.-Y.; Choi, J.-W.; Kim, H. Determination of hand surface area by sex and body shape using alginate. *J. Physiol. Anthropol.* **2007,** *26*, 475-483. CrossRef: http://dx.doi.org/10.2114/jpa2.26.475; PubMed: https://www.ncbi.nlm.nih.gov/pubmed/17704626.
2. Wiertlewski, M.; Hayward, V. Mechanical behavior of the fingertip in the range of frequencies and displacements relevant to touch. *J. Biomech.* **2012,** *45*, 1869-1874. CrossRef: http://dx.doi.org/10.1016/j.jbiomech.2012.05.045; PubMed: https://www.ncbi.nlm.nih.gov/pubmed/22732906.
3. Gao, X. *Relative effectiveness of ventilation in community indoor environmentsfor controlling infection*. The University of Hong Kong: Pokfulam, Hong Kong, 2011.
4. Atkinson, M. P.; Wein, L. M. Quantifying the routes of transmission for pandemic influenza. *Bull. Math. Biol.* **2008,** *70*, 820-867. CrossRef: http://dx.doi.org/10.1007/s11538-007-9281-2; PubMed: https://www.ncbi.nlm.nih.gov/pubmed/18278533.
5. Lopez, G. U. *Transfer of Microorganisms from Fomites to Hands and Risk Assessment of Contaminated and Disinfected Surfaces.* The University of Arizona: Tucson, Arizona, USA, 2013.

6. Rusin, P.; Maxwell, S.; Gerba, C. Comparative surface-to-hand and fingertip-to-mouth transfer efficiency of gram-positive bacteria, gram-negative bacteria, and phage. *J. Appl. Microbiol.* **2002,** *93*, 585-592. CrossRef: http://dx.doi.org/10.1046/j.1365-2672.2002.01734.x; PubMed: https://www.ncbi.nlm.nih.gov/pubmed/12234341.
7. Lopez, G. U.; Gerba, C. P.; Tamimi, A. H.; Kitajima, M.; Maxwell, S. L.; Rose, J. B. Transfer efficiency of bacteria and viruses from porous and nonporous fomites to fingers under different relative humidity conditions. *Appl. Environ. Microbiol.* **2013,** *79*, 5728-5734. CrossRef: http://dx.doi.org/10.1128/AEM.01030-13; PubMed: https://www.ncbi.nlm.nih.gov/pubmed/23851098.
8. Lee, J.; Zoh, K.; Ko, G. Inactivation and UV disinfection of murine norovirus with TiO2 under various environmental conditions. *Appl. Environ. Microbiol.* **2008,** *74*, 2111-2117. CrossRef: http://dx.doi.org/10.1128/AEM.02442-07; PubMed: https://www.ncbi.nlm.nih.gov/pubmed/18245239.
9. Mokhtari, A.; Jaykus, L.-A. Quantitative exposure model for the transmission of norovirus in retail food preparation. *Int. J. Food Microbiol.* **2009,** *133*, 38-47. CrossRef: http://dx.doi.org/10.1016/j.ijfoodmicro.2009.04.021; PubMed: https://www.ncbi.nlm.nih.gov/pubmed/19450890.
10. Cannon, J. L.; Papafragkou, E.; Park, G. W.; Osborne, J.; Jaykus, L.-A.; Vinjé, J. Surrogates for the study of norovirus stability and inactivation in the environment: a comparison of murine norovirus and feline calicivirus. *J. Food Prot.* **2006,** *69*, 2761-2765. CrossRef: http://dx.doi.org/10.4315/0362-028X-69.11.2761; PubMed: https://www.ncbi.nlm.nih.gov/pubmed/17133824.
11. Kwok, Y. L. A.; Gralton, J.; McLaws, M.-L. Face touching: A frequent habit that has implications for hand hygiene. *Am. J. Infect. Control.* **2015,** *43*, 112-114. CrossRef: http://dx.doi.org/10.1016/j.ajic.2014.10.015.; PubMed: https://www.ncbi.nlm.nih.gov/pubmed/25637115.
12. Elder, N. C.; Sawyer, W.; Pallerla, H.; Khaja, S.; Blacker, M., Hand hygiene and face touching in family medicine offices: a Cincinnati Area Research and Improvement Group (CARInG) network study. *J. Am. Board Fam. Med.* **2014,** *27*, 339-346. CrossRef: http://dx.doi.org/10.3122/jabfm.2014.03.130242; PubMed: https://www.ncbi.nlm.nih.gov/pubmed/24808112.
13. Marks, P.; Vipond, I.; Carlisle, D.; Deakin, D.; Fey, R.; Caul, E. Evidence for airborne transmission of Norwalk-like virus (NLV) in a hotel restaurant. Epidemiol. Infect. 2000, 124, 481-487. CrossRef: http://dx.doi.org/10.1017/s0950268899003805; PubMed: https://www.ncbi.nlm.nih.gov/pubmed/10982072.
14. Chan, M. C.; Sung, J. J.; Lam, R. K.; Chan, P. K.; Lee, N. L.; Lai, R. W.; Leung, W. K. Fecal viral load and norovirus-associated gastroenteritis. Emerg. Infect. Dis. 2006, 12, 1278. CrossRef: http://dx.doi.org/10.3201/eid1208.060081; PubMed: https://www.ncbi.nlm.nih.gov/pubmed/16965715.
15. Thebault, A.; Teunis, P. F. M.; Le Pendu, J.; Le Guyader, F. S.; Denis, J.-B. Infectivity of GI and GII noroviruses established from oyster related outbreaks. Epidemics. 2013, 5, 98-110. CrossRef: http://dx.doi.org/10.1016/j.epidem.2012.12.004; PubMed: https://www.ncbi.nlm.nih.gov/pubmed/23746803.
16. Pittet, D.; Dharan, S.; Touveneau, S.; Sauvan, V.; Perneger, T. V. Bacterial contamination of the hands of hospital staff during routine patient care. Arch. Intern. Med. 1999, 159, 821-826. CrossRef: http://dx.doi.org/10.1001/archinte.159.8.821; PubMed: https://www.ncbi.nlm.nih.gov/pubmed/10219927.
